# Nonsteroidal Anti-inflammatory Drug (NSAID)-Induced Enteropathy Mimicking Crohn’s Disease: A Reversible Cause of Ileitis

**DOI:** 10.7759/cureus.108296

**Published:** 2026-05-05

**Authors:** Justin Baik, Scott McGuire, Neil Hughes, Tooba Tariq

**Affiliations:** 1 Internal Medicine, Western Michigan University Homer Stryker M.D. School of Medicine, Kalamazoo, USA; 2 Gastroenterology, Bronson Methodist Hospital, Kalamazoo, USA

**Keywords:** crohn’s disease mimic, fecal calprotectin, medication review, nsaid enteropathy, terminal ileitis

## Abstract

A 77-year-old man with chronic iron-deficiency anemia and prior small bowel resection presented with terminal ileal ulcerations suggestive of Crohn’s disease, along with elevated C-reactive protein and markedly increased fecal calprotectin. Medication review revealed chronic meloxicam use. Following discontinuation, his inflammatory markers and anemia normalized, and repeat colonoscopy demonstrated complete resolution of ileal ulcers. This case highlights nonsteroidal anti-inflammatory drug (NSAID)-induced enteropathy as an important mimic of Crohn’s disease and emphasizes the importance of thorough medication review and clinical correlation to prevent misdiagnosis and unnecessary immunosuppressive therapy.

## Introduction

Nonsteroidal anti-inflammatory drugs (NSAIDS) are among the most commonly used medications worldwide and are well recognized for their potential gastrointestinal (GI) toxicity. While upper GI complications such as peptic ulcer disease and gastritis are widely known, the distal small bowel is also an underrecognized site of NSAID-induced injury [1]. Current literature shows that distal GI complications are becoming increasingly more common; in fact, they comprise as high as 40% of all NSAID-induced adverse GI side-effects [2].

The presentation and findings of NSAID-induced enteropathy can closely resemble those of Crohn’s disease, particularly when ulcerations are localized to the terminal ileum. Both conditions may present with iron-deficiency anemia, elevated inflammatory markers, and small bowel ulceration, making differentiation challenging [3]. However, the clinical course and response to withdrawal of the offending agent can be critical in distinguishing between these two entities.

This case highlights the diagnostic dilemma of differentiating Crohn’s disease from NSAID-induced small bowel injury in an elderly patient presenting with chronic iron-deficiency anemia and terminal ileal ulcerations.

## Case presentation

A 77-year-old male with a history of osteoarthritis, chronic iron-deficiency anemia (diagnosed four months prior to presentation) and prior small bowel obstruction status-post small bowel resection in 2022 presented for further evaluation of his anemia. Six weeks prior to presentation, he received an intravenous iron infusion after several years of oral iron supplementation.

The patient denied any GI symptoms like abdominal pain, nausea, vomiting, early satiety, unintentional weight loss, melena, hematochezia, or diarrhea. He reported no recent changes in appetite or bowel habits apart from intermittent constipation related to his iron therapy.

Laboratory evaluation in December 2024 demonstrated persistently low iron levels despite intravenous replacement, with an iron level of 40 µg/dL (reference: 60-170 µg/dL), iron saturation of 19% (reference: 20-50%), and total iron-binding capacity (TIBC) of 208 µg/dL (reference: 240-450 µg/dL). Ferritin was within normal limits (reference: 30-400 ng/mL). Complete blood count revealed a hemoglobin of 11.3 g/dL (reference: 13.5-17.5 g/dL), mean corpuscular volume of 96 fL (reference: 80-100 fL), and a red cell distribution width of 14.4% (reference: 11.5-14.5%), with a normal platelet count of 194 x 10⁹/L (reference: 150-450 x 10⁹/L) (Tables 1-3).

**Table 1:** Labs CBC: complete blood count, RBC: red blood cell, WBC: white blood cell, MCV: mean corpuscular volume, MCH: mean corpuscular hemoglobin, MCHC: mean corpuscular hemoglobin concentration, RDW: red blood cell distribution width, MPV: mean platelet volume

CBC	Patient Value	Normal Range
WBC	7.2	4.0-10.5x10³/µL
RBC	3.57	4.2-5.4x10⁶/µL
Hemoglobin	11.3	12-16g/dL
Hematocrit	34.4	36-46%
MCV	96.4	80-100 fL
MCH	31.7	27-33 pg
MCHC	32.8	32-36 g/dL
RDW	14.4	11-15 %
Platelets	194	150-450x10³/µL
MPV	8.7	7.5-11.5fL

**Table 2:** Labs continued TIBC: total iron-binding capacity

Iron Studies	Patient Value	Normal Range
Serum iron	40	60-170 µg/dL
Ferritin	367	30-400 ng/mL
Transferrin Saturation	19	20-50%
Transferrin	164	200-360 mg/dL
TIBC	208	240-450 µg/dL
Folate	56	3-17 ng/mL

**Table 3:** Labs continued CRP: C-reactive protein

Other	Patient Value	Normal Result/Range
Fecal calprotectin	6103	<80 µg/g
CRP	41.5	<6.0 mg/L

Review of prior endoscopic records revealed a colonoscopy performed four years ago that demonstrated aphthous ulcers in the terminal ileum. Given his history of chronic iron-deficiency anemia, previous small bowel resection, and colonoscopic evidence of terminal ileal ulceration, there was growing concern for underlying small bowel pathology as a potential source of chronic blood loss or inflammation, with inflammatory bowel disease (IBD) being high on the differential.

**Figure 1:**
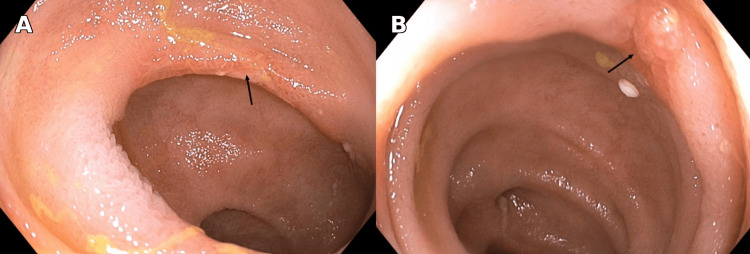
Multiple superficial ulcers in the terminal ileum surrounding inflammatory mucosa with no hemorrhage; performed cold forceps biopsy. A. Colonoscopic view showing a superficial ulcer in the terminal ileum with an arrow indicating the lesion. B. Colonoscopic view of a different superficial ulcer in the terminal ileum with an arrow indicating the lesion.

**Figure 2:**
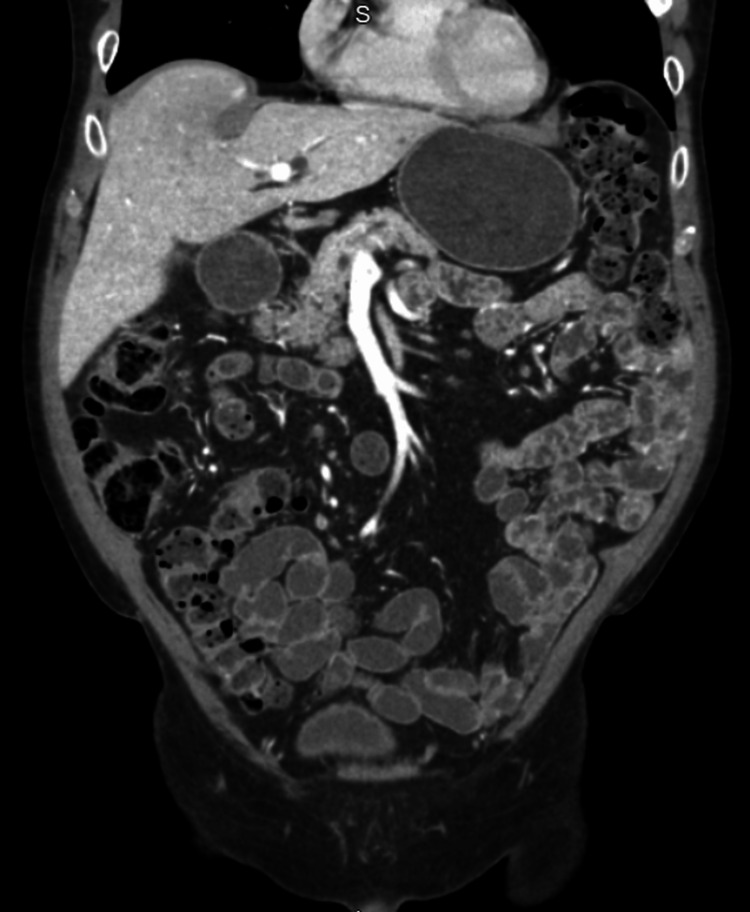
CT Enterography shows no acute inflammatory changes or fluid collections noted within the abdomen or pelvis, or any significant mucosal thickening or secondary signs to suggest Crohn's enteritis.

Therefore, further investigation revealed a markedly elevated C-reactive protein (CRP) of 41.5 mg/L (reference: <5 mg/L) and a fecal calprotectin level of 6103 µg/g (reference: <50 µg/g), suggestive of active intestinal inflammation. A colonoscopy demonstrated multiple small, superficial ulcers in the terminal ileum with surrounding inflammatory mucosa (Figure 1). Colon biopsies were negative for inflammation or microscopic colitis. Small intestinal biopsies of the terminal ileum showed fragments of small bowel and colonic mucosa with focal mild active inflammation with few neutrophils in the lamina propria and superficial epithelium. There were changes of chronic crypt injury and focal mucosal granuloma. An upper endoscopy with endoscopic ultrasound (EUS) performed the same month demonstrated mild gastritis and duodenal diverticulosis. CT enterography demonstrated no inflammation, strictures or secondary findings to suggest Crohn’s enteritis, contrary to the colonoscopy findings (Figure 2).

Given the significantly elevated fecal calprotectin, elevated CRP and the presence of distal small bowel ulcers, there was concern for Crohn’s disease. However, the patient was noted to be taking 15mg meloxicam once a day for almost a year, an NSAID known to cause small bowel ulceration and inflammation. Of note, he was not on any acid suppression therapy. He was advised to discontinue meloxicam, and his inflammatory markers and anemia were closely monitored. Other causes of ileitis such as infection and celiac disease were also considered, but tests were not pursued due to high degree of suspicion of NSAID-induced enteropathy.

Following discontinuation of meloxicam, the patient demonstrated significant clinical and laboratory improvement during his follow-up three months later. His inflammatory markers normalized, his hemoglobin increased from 12.4 g/dL to 13 g/dL, and his fecal calprotectin decreased dramatically from 6103 µg/g to 1474 µg/g. Serum iron improved from 48 µg/dL to 78 µg/dL, iron saturation normalized at 33%, and ferritin remained stable (Table 4).

**Table 4:** Lab improvements after resolution CRP: C-reactive protein

	Initial Value	After Resolution	Normal Range
Hemoglobin	11.3	13.0	12-16g/dL
Serum iron	40	78	60-170 µg/dL
Transferrin Saturation	19	28	20-50%
Transferrin	164	191	200-360 mg/dL
CRP	41.5	<3.0	<6.0 mg/L
Fecal calprotectin	6103	1474	<80 µg/g

**Figure 3:**
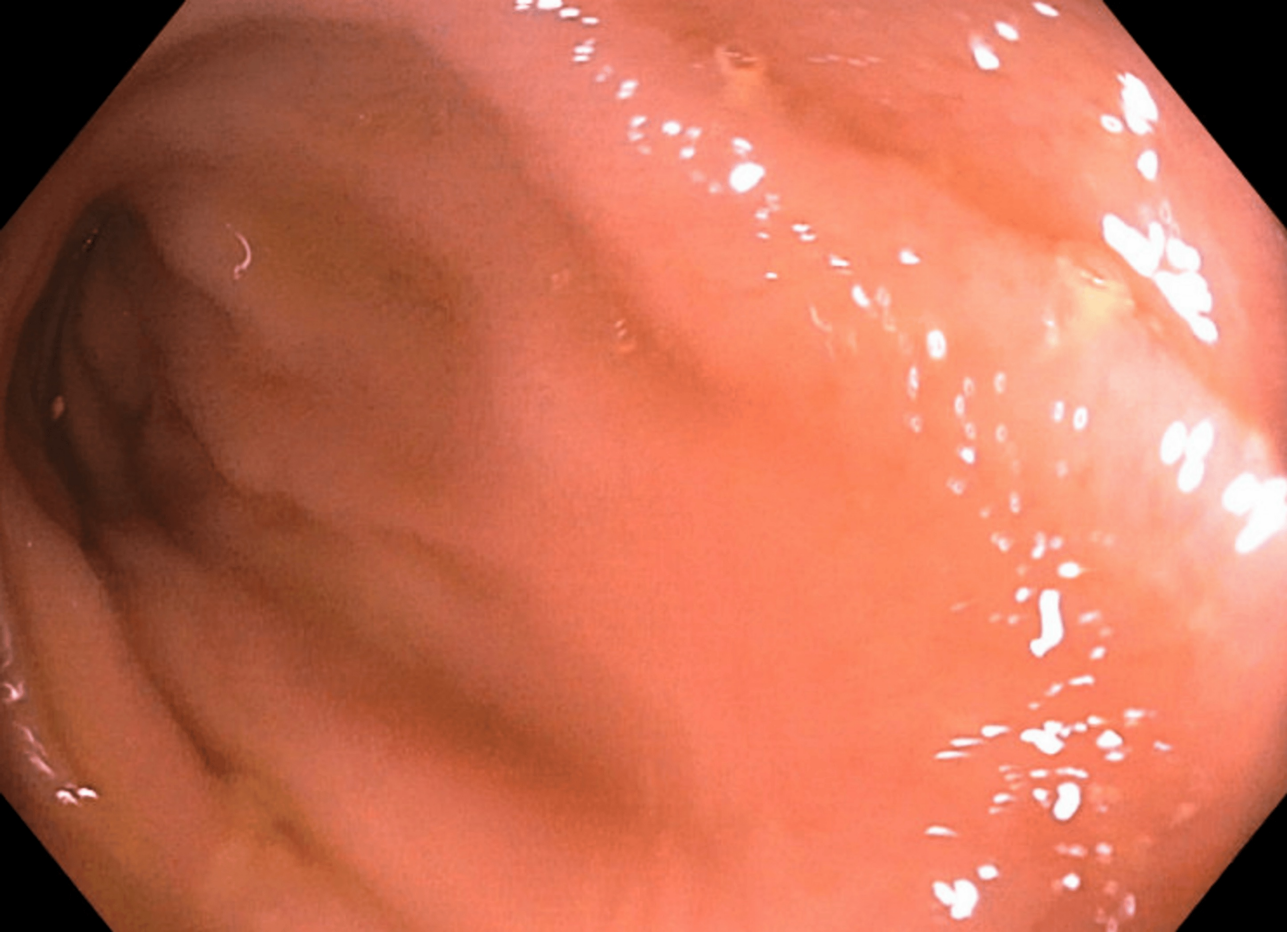
Repeat colonoscopy nine months after meloxicam discontinuation. The terminal ileum appeared normal. No ulcers noted.

Clinically, he reported regular, formed bowel movements without constipation or diarrhea and denied abdominal pain, melena, or hematochezia. A repeat colonoscopy seven months following cessation of meloxicam demonstrated complete resolution of the previously observed ulcers, with a normal-appearing terminal ileum and no evidence of active inflammation (Figure 3).

The overall clinical course suggested that the patient’s small bowel ulcers and inflammation were secondary to NSAID-induced enteropathy rather than Crohn’s disease. Discontinuation of meloxicam led to normalization of inflammatory markers, improvement in anemia, and endoscopic healing of the terminal ileum. Careful medication review and observation following drug cessation can prevent unnecessary initiation of immunosuppressive therapy.

## Discussion

NSAID-induced enteropathy is an increasingly recognized cause of small bowel ulceration and anemia, often mimicking Crohn’s disease. The pathogenesis involves inhibition of cyclooxygenase (COX) enzymes, leading to reduced prostaglandin synthesis and disruption of the mucosal barrier [4]. NSAIDs also increase intestinal permeability by impairing mitochondrial function, allowing bile acids and bacterial toxins to penetrate the mucosa and initiate inflammation [5].

Clinically, NSAID enteropathy may present with iron-deficiency anemia, occult gastrointestinal bleeding, or nonspecific abdominal symptoms. Endoscopically, injury in the terminal ileum typically demonstrates discrete erosions or sharply demarcated shallow ulcers, rather than diffuse inflammation. Small bowel diaphragm disease is a NSAID-induced small bowel obstruction that is pathognomonic of long-term NSAID use [6]. Histologic evaluation may reveal nonspecific acute inflammation and mucosal erosion, as with our case. A study by Nguyen et al. showed that decreased lamina propria lymphoplasmacytic inflammation was the most specific, and the presence of gland atrophy was the most sensitive feature of NSAID enteropathy [7]. Meanwhile, in Crohn’s disease, the most specific finding of epithelioid granulomas is seen in fewer than 20% of biopsies, and additional histologic features may include focal patchy inflammatory infiltrates, lymphoid aggregates, and pyloric gland metaplasia, underscoring the importance of clinical context [8].

Complications of NSAID-related lower GI injury remain a significant clinical concern, as they are frequently overlooked or misinterpreted as inflammatory bowel disease. Failure to recognize this entity may lead to unnecessary immunosuppressive therapy, invasive procedures, or avoidable morbidity. Early identification and prompt withdrawal of the offending agent are therefore critical for optimal outcomes [9]. Current guidelines emphasize discontinuing NSAID use and reassessing inflammatory markers before diagnosing inflammatory bowel disease [10].

## Conclusions

In conclusion, this case shows normalization of the patient’s CRP and fecal calprotectin levels, along with resolution of ileal ulcers following meloxicam cessation. This case underscores the importance of considering NSAID-induced enteropathy in patients with terminal ileal ulcerations and chronic iron-deficiency anemia. The patient’s complete clinical, biochemical, and endoscopic recovery following discontinuation of meloxicam adds to the growing literature on how closely NSAID injury can mimic Crohn’s disease and demonstrates the value of medication review before initiating immunosuppressive therapy.
